# Enhancing of Wound Healing in Burn Patients through *Candida albicans* β-Glucan

**DOI:** 10.3390/jof8030263

**Published:** 2022-03-04

**Authors:** Fateme Abedini, Shahla Roudbar Mohammadi, Mostafa Dahmardehei, Marjan Ajami, Maryam Salimi, Halala Khalandi, Monireh Mohsenzadegan, Farhad Seif, Bahador Nikoueian Shirvan, Sanaz Yaalimadad, Maryam Roudbary, Célia F. Rodrigues

**Affiliations:** 1 Department of Immunology, School of Medicine, Iran University of Medical Sciences, Tehran 1449614535, Iran; fateme.abediny@gmail.com; 2 Department of Mycology, Faculty of Medical Sciences, Tarbiat Modares University, Tehran 14115-111, Iran; sh.mohammadi@modares.ac.ir (S.R.M.); bahador_nikoueian@yahoo.com (B.N.S.); s.yam13731994@gmail.com (S.Y.); 3 Department of Plastic and Reconstructive Surgery, Burn Research Center, Iran University of Medical Sciences, Tehran 14115-111, Iran; m.dahmardehei@gmail.com; 4 Department of Food and Nutrition Policy and Planning, National Nutrition and Food Technology Research Institute, School of Nutrition Sciences and Food Technology, Shahid Beheshti University of Medical Science, Tehran 19395-4741, Iran; marjan.ajami80@gmail.com; 5 Department of Medical Mycology, School of Medicine, Mazandaran University of Medical Sciences, Sari 33971-48157, Iran; maryamsalimita1992@gmail.com; 6 Department of Parasitology and Mycology, School of Medicine, Iran University of Medical Sciences, Tehran 1449614535, Iran; halala.khalandi@yahoo.com; 7 Department of Medical Laboratory Sciences, Faculty of Allied Medical Sciences, Iran University of Medical Sciences, Tehran 1449614535, Iran; monirehmohsenzadegan@gmail.com; 8 Department of Immunology and Allergy, Academic Center for Education, Culture and Research (ACECR), Tehran 1315795613, Iran; farhad.seif@outlook.com; 9 LEPABE—Laboratory for Process Engineering, Environment, Biotechnology and Energy, Faculty of Engineering, University of Porto, 4200-465 Porto, Portugal; 10 TOXRUN—Toxicology Research Unit, Cooperativa de Ensino Superior Politécnico e Universitário—CESPU, 4585-116 Gandra, Portugal

**Keywords:** β-glucan, wound healing, burn patients, cytokine assay, *Candida albicans*, ointment

## Abstract

The mortality and disability-adjusted life years (DALYs) of burn patients are decreasing over time. However, finding novel effective treatment approaches using natural agents is highly considered to reduce the burden of burn injuries. One of the recent agents used in wound healing is β-glucan, mainly extracted from fungi cell walls. This study aimed to evaluate the effect of 5% (m/m) of yeast β-glucan ointment on burn wound healing and to assess the impact of β-glucan on cytokines during the treatment. Thirty-three patients with second or third-degree burns were enrolled in this study. Two groups of twenty-three and ten patients used yeast 5% (m/m) β-glucan ointment (study group) and Stratamed ointment (control), respectively, on a daily basis, for a maximum of four weeks. The size of the burn wounds was measured before and at the end of the treatment. Blood samples of 14 and 10 patients in the β-glucan and control groups, respectively, were obtained before and after the treatment, and the enzyme-linked immunosorbent assay (ELISA) was performed to measure the serum concentration of the IL-4, IL-17, and IFN-γ cytokines. The log-binomial model was used to assess the efficacy of the β-glucan ointment on burn wound healing. ANOVA/ANCOVA was employed to assess the effects of β-glucan on the serum concentration of cytokines. After adjusting for potential confounders/covariates, patients receiving β-glucan had better wound healing (RR = 4.34; 95% CI: 0.73 to 25.67; *p* = 0.11). There was a significant difference in IL-4 secretion between the β-glucan and control groups after adjusting for potential confounders/covariates (MD = 77.27; 95% CI: 44.73 to 109.82; *Cohen’s d* = 2.21; 95% CI: 1.16 to 3.24; *p* = 0.0001). The results indicate that 5% (m/m) of β-glucan has efficacy in burn wound healing, and a significant difference was found in the level of IL-4 after receiving β-glucan. Further studies with a two-arm design and long-term use of ointment are needed to confirm the effect of β-glucan on wound healing and cytokine secretion.

## 1. Introduction

According to the global burden of disease reports, the age-standardized incidence and mortality rate of burns in 2017 were 119 and 1.6 per 100,000, representing a decline of 5.4% and 46.6% from 1990 to 2017, respectively. Furthermore, the disability-adjusted life years (DALYs) decreased by 43.7% between 1990 and 2017 [1]. The decrease in death rate and DALYs are attributed to therapeutic approaches, including antimicrobial agents, various ointments, skin grafts, and cell therapy for severe burn wounds [2]. Still, one study in Spain estimated that the annual cost of burn-related hospitalization and mortality was USD 99,773 [3]. Therefore, discovering effective treatments with low cost can be beneficial, and β-glucan is a therapeutic agent recently used to facilitate wound healing.

β-glucans are polysaccharides isolated from the cell walls of fungi (e.g., yeast), bacteria, or mushrooms. Various types of β-glucans have different bioactivities, depending on solubility and molecular weight. The higher weight and soluble ones have the highest activity level, and soluble β-glucans are stronger immunostimulators [4]. Several receptors can recognize β-glucans, including dectin-1 and complement receptor 3 (CR3), expressed on various cells, such as natural killer (NK) cells, dendritic cells (DCs), monocytes, and macrophages. Burn-induced tissue damage is mainly caused by the overproduction and diffusion of reactive oxygen species (ROS) and active inflammatory reactions induced by different cell types among molecular mediators activated in tissue damage caused by ischemia or protective signal pathways. Mitochondrial KATP-sensitive potassium channels (ATP) [5,6] and cytokine production via dectin-1 are induced through two pathways: the Syk-dependent pathway that produces T helper 2 (Th2) type anti-inflammatory cytokines such as interleukin-10 (IL-10), and the Toll-like receptor-Myd88, which induces pro-inflammatory cytokines such as tumor necrosis factor-α (TNF-α) [7]. The interaction between β-glucan and its receptors can also activate nuclear factor-κB (NF-κB) and activator protein-1 (AP-1) transcription factors. Therefore, it can initiate several innate and adaptive responses, including activating via neutrophil and monocyte, complement activation, cytokine secretion, and T cell-mediated cytotoxicity [4,8]. Moreover, it can activate cellular immunity via macrophage stimulation, protect against infections, and affect wound healing [8,9,10].

Wound healing has three phases. The first phase is inflammation, in which neutrophils and macrophages immigrate to the wound and remove the bacteria; the second phase is proliferation, in which fibroblasts play a crucial role, and the extracellular matrix is renewed; and, finally, in the third phase—remodeling—blood flow and the number of cells in the wound area decrease [11]. Two responses occur in the inflammation phase: pro-inflammatory and transient anti-inflammatory [11]. Interferon-gamma (IFN-γ) is a pro-inflammatory cytokine that enhances collagenase expression, decreases collagen synthesis, and declines re-epithelialization; however, non-stop expression of IFN-γ can be harmful [11,12]. Interleukin-17 (IL-17) is another pro-inflammatory cytokine that is increased during burn injuries [13]. Some cytokines limit the pro-inflammatory response and trigger the proliferation phase of healing, thereby playing essential roles in wound repair [11]. An increased level of pro-inflammatory cytokines in non-healing wounds inhibits progression to the next stage and impairs wound healing [14]. Interleukin-4 (IL-4), as an anti-inflammatory cytokine secreted from mast cells (MCs), induces fibroblast activities and leads to wound repair [15].

Several studies have shown the effects of β-glucan on wound healing in animals and humans. This is the case in a recent observational study on soluble β-glucan gel effects on various wounds, demonstrating the significant healing efficacy of this polymer [16]. Additionally, three clinical trial studies elucidated the potent efficacy of β-glucan in lessening the wound size, shortening the treatment period, and reducing the treatment cost [17,18,19]. Although some studies were conducted to indicate the effect of β-glucan in cytokine secretion in different diseases, such as cancer and allergy [20,21], there is no study to evaluate the effect of β-glucan in cytokine secretion in patients with burn injuries.

For the first time (to our knowledge), this observational study aimed to evaluate the association between soluble β-glucan, extracted from *Saccharomyces cerevisiae* cell wall, and healing in burn injuries through morphological assessment. A secondary goal was to assess the effect of topical β-glucan usage on the secretion of systemic pro-inflammatory (IFN-γ and IL-17) and anti-inflammatory (IL-4) cytokines.

## 2. Material and Methods

### 2.1. Patients

Thirty-three patients and ten patients as a control group with burn wounds, referred to Mottahari Burns Hospital affiliated to Iran University of Medical Sciences (Tehran, Iran), were enrolled in this study from October 2018 to September 2019. The inclusion criteria for the two groups were both genders ≥25 years old with a second or third-degree burn, no previous local anti-fungal therapy, and burn size more than 10 cm^2^. Case patients received 5% (m/m) β-glucan ointment dissolved in petrolatum (Petrolatum Ointment Base, Ehsan Chemi Company, Tehran, Iran), and the control group received Stratamed ointment as a topical form in those subjects. Indeed, 100% natural yeast β-glucan powder from *Saccharomyces cerevisiae* (light Yellow Fine powder) was purchased (CAS NO-9012-72-0, China) and used for ointment preparation. Case subjects who received immunosuppressive drugs or immunocompromised patients, patients with acute infections (e.g., pneumonia, tuberculosis) and pregnant women were excluded from the study to avoid probable side effects of beta-glucan. Participants used the synthesized 5% (m/m) β-glucan ointment in the case group and Stratamed ointment in the control group daily for 1, 2, 3, or 4 weeks based on remission of wound healing and were followed up during the study for the wound size. Moreover, patients in the control group routinely received the standard treatment of wound healing according to national guidelines including debridement of wounds and Stratamed ointment. The wound size was measured at the first visit before using the ointments, and finally, after the last day of ointment administration in both case and control groups. Informed consent forms were obtained from all participants at the beginning of the treatment. The Research Ethics Committee of Iran University of Medical Sciences approved the study (NO. IR.IUMS.REC 1394.26610). The names and private information of the patients were kept confidential.

### 2.2. Blood Sample Collection of Patients

Approximately 4 mL of coagulated blood was obtained from each patient in both groups at two different times, the first one at the beginning of the study before using the ointment and the second one after the last day of ointment administration to evaluate cytokine assay (14 and 10 patients in the β-glucan group and control group participated in this step, respectively). The samples were centrifuged at 1000× *g* for 10 min to obtain serum. The sera were aliquoted in microtubes and stored at −80 °C.

### 2.3. Cytokine Assay

According to the manufacturer’s protocol, the serum levels of IL-4, IL-17 and IFN-γ were measured by commercial sandwich ELISA kits (IBL, Germany). The intra-assay coefficients of variation (CVs) for IL-4, IL-17, and IFN-γ were 4.8%, 7.1%, and 4.5%, respectively. The inter-assay CVs for the above cytokines were 5.6%, 9.1%, and 5.7%.

### 2.4. Statistical Analysis

The study’s primary outcome was wound healing, measured as reducing wound size precisely after the last day of the treatment, categorized into complete and partial wound healing. The log-binomial model was employed to compare wound healing in two groups; potential confounders/covariates were also adjusted. Risk ratio (RR) and 95% confidence interval (CI) were reported to show the strength of the relationship. ANOVA/ANCOVA was performed to assess differences in systemic inflammatory and anti-inflammatory cytokines (IL-17, IFN-γ, and IL-4) and adjust for baseline levels of cytokines and potential confounders/covariates. The standardized mean difference (SMD) based on *Cohen’s d* and its corresponding 95% CI were used to report the magnitude of the effect. All statistical tests were two-tailed, and *p* ≤ 0.05 was considered statistically significant. The analyses were performed using Stata software version 14 (StataCorp LP, College Station, TX, USA). All the statistical analyses and reports of the results were adapted to the Statistical Analyses and Methods in the Published Literature (SAMPL) guidelines [22].

## 3. Results

### 3.1. Participants’ Demographics and Baseline Characteristics and Wound Healing Evaluation

Thirty-three patients receiving β-glucan ointment and ten receiving Stratamed ointment as a control group, with the median age of 47 and 45, respectively, were enrolled in this study (Table 1).

According to Table 2, the crude RR illustrated a marginally significant effect of β-glucan ointment on wound healing (RR = 5.65; 95% CI: 0.85, 37.55; *p* = 0.07); however, RD showed a significant difference between the probability of remission in patients receiving β-glucan ointment and those receiving Stratamed ointment. When this effect was adjusted for ointment usage duration (<2 weeks and ≥2 weeks), wound duration (<1 week, 1–2 weeks, ≥2 weeks), and burn degree (degree 2 and degree 3), the RR decreased and showed a non-significant effect of β-glucan on burn treatment (RR = 4.34; 95% CI: 0.73, 25.67; *p* = 0.11), but the RD still showed a significant difference (RD = 0.55; 95% CI: 0.24, 0.87; *p* = 0.001) (Table 2).

### 3.2. Differences in Serum Levels of IL-4, IL-17, and IFN-γ after Treatment

The serum levels of the cytokines were measured in 14 patients receiving β-glucan ointment and 10 who used Stratamed ointment in the control group by ELISA before and after the treatment. IL-4 concentration in patients using β-glucan was higher than the other group (MD = 75.48; 95% CI: 48.88 to 102.08; *Cohen’s d* = 2.44; 95% CI: 1.34 to 3.51; *p* < 0.0001); by adjustment for ointment usage duration, wound duration, and burn degree, this difference was also significant (MD = 77.27; 95% CI: 44.73 to 109.82; *Cohen’s d* = 2.21; 95% CI: 1.16 to 3.24; *p* = 0.0001). There were no significant differences in IL-17 and IFN-γ between these two groups (Table 3).

## 4. Discussion

Although the mortality and DALYs of burn patients have decreased in recent years, attempts to find the best treatment are still ongoing. Numerous studies have evaluated the efficacy of β-glucan in wound healing [16,23]. However, the effect of β-glucan on cytokines in patients’ burn injuries remains unclear.

This study aimed to assess the relationship between 5% (m/m) β-glucan ointment usage and remission of burn wounds compared with Stratamed ointment usage in the control group and evaluate the association between the two treatments and serum concentration of inflammatory/anti-inflammatory cytokines.

Our findings demonstrate that patients using 5% (m/m) β-glucan ointment had about 5.5 times more probability of complete wound healing, showing a strong relationship between β-glucan and healing. The adjustment for potential confounders diluted the relationship (4.3 times more probability of wound healing); yet, this result still suggests a strong association. In 2018, Hunt and colleagues assessed the effectiveness of bioactive soluble β-glucan gel (BSBG) in healing static chronic wounds (with any etiology) [16]. The authors selected patients who received only the standard care (control group) and patients who received standard care plus BSBG twice a week (BSBG group). After 12 weeks, 81% of patients (116/144) in the BSBG group were healed completely, compared with 66% of patients (91/138) in the control group. Our results showed that 57% of the patients (13/23) had completely healed after the β-glucan ointment therapy compared to 10% (1/10) in the control group. Another study evaluated the effects of soluble β-glucan (SBG) on static wounds [18]. Participants had various hard-to-heal wounds in this study and received SBG twice a week for at least four weeks. The results demonstrated that participants who used the SBG for more than four weeks had more reduction in wound size, and more than 50% reduction in wound sizes occurred in 58% of patients (15/26) at week 12.

The present study showed a more potent effect of β-glucan in wound healing, which might be related to the fact that, in our study, wounds were not difficult to heal, and β-glucan can further positively affect younger wounds. Furthermore, this study showed that the mean concentrations of pro-inflammatory cytokines (IL-17 and IFN-γ) had no significant difference after using the β-glucan or Stratamed ointment. However, there is a large and conclusive association between using β-glucan ointment and the increment in IL-4 (an anti-inflammatory cytokine) secretion, possibly because IL-4 is one of the cytokines secreted from Th2 cells. Furthermore, it has been shown that *C. albicans* β-glucan can induce the production of Th2 anti-inflammatory cytokines through the dectin-1 receptor [7], which is supported by our finding. Correspondingly, another study reported a dose-dependent effect of β-glucan on IL-17A and IFN-γ induction [24], which was also related to higher treatment duration. The same report indicated that the ointment usage (per day) could affect the results and express this relationship more accurately.

The use of different pharmaceutical preparations with β-glucan and β-glucan from different fungi species has also been addressed. In 2018, Yasuda et al. evaluated the therapeutic effects of a film dressing β-glucan paramylon in wound healing in mice and assessed the cytokine changes after using the paramylon film. They concluded that mice with paramylon film significantly decreased serum concentration of IFN-γ [25]. Our findings showed no differences in levels of IFN-γ at baseline and after treatment with β-glucan ointment, plausibly due to the difference in the type of β-glucan implementation.

In addition, Lee et al. compared the effects of β-glucan (extracted from *Agrobacterium* sp. R259) capsules on activating NK cells in healthy adults. Serum levels of cytokines, including IFN-γ, were measured at the baseline and after the eight weeks that the participants received the capsule. The results indicated no significant changes in serum concentrations of IFN-γ in participants receiving the β-glucan capsule. Moreover, no significant differences were observed in the above cytokines between the β-glucan and placebo [26], which is in agreement with our findings. Naturally, this work has some limitations. First, this is an observational study with restrictions on showing the efficacy of the ointment, including numbers of confounders or covariates that affect the results; second, a larger sample size of the study could bring extra promising findings regarding the efficacy of β-glucan.

## 5. Conclusions

In general, RR showed a strong relationship between β-glucan usage and wound healing. Furthermore, *Cohen’s d* showed a considerable effect of β-glucan on IL-4 secretion, indicating the role of β-glucan on the function of Th2 cells. However, there were no significant changes in IFN-γ and IL-17 secretion after using the β-glucan ointment, probably due to the wound burn conditions and small sample size, which can affect the β-glucan characteristics. A two-arm, randomized clinical trial study with a larger sample size and more prolonged treatment duration can further elucidate the effect of β-glucan on cytokines in burn patients.

## Figures and Tables

**Table 1:** Baseline characteristics of the participants.

Baseline Characteristics	Receiving β-Glucan (*n* = 23)	Receiving Stratamed (*n* = 10)
***Age:*** **Median [Q1–Q3]**	47 (36–55)	45 (41–53)
***Burn size*** **(cm^2^):** **Median [Q1–Q3]**	108 (46.5–182)	104.5 (21–144)
***Sex: n*** **(%)** **Male** **Female**	13 (56.5) 10 (43.5)	5 (50) 5 (50)
***Burn degree: n*** **(%)** **Second-degree** **Second/third degree** **Third-degree**	12 (52.2) 3 (13.0) 8 (34.8)	8 (80.0) 0 (0.0) 2 (20.0)
***Wound duration: n*** **(%)** **<1 week** **1–2 weeks** **2 weeks** **>2 weeks**	2 (8.7) 9 (39.1) 8 (34.8) 4 (17.4)	2 (20.0) 5 (50.0) 3 (30.0) 0 (0.0)
***Burn location: n*** **(%)** **Arm and hand** **Leg and foot** **Others (head, neck, face, etc.)**	12 (52.2) 8 (34.8) 3 (13.0)	7 (70.0) 3 (30.0) 0 (0.0)
***Ointment usage: n*** **(%)** **1 week** **2 weeks** **3 weeks** **4 weeks**	5 (21.7) 6 (26.1) 10 (43.5) 2 (8.7)	4 (40.0) 6 (60.0) 0 (0.0) 0 (0.0)

**Table 2:** The effectiveness of β-glucan ointment on wound healing.

Models	RR (95% CI)	*p*	RD (95% CI)	*p*
**Model A ^a^**	5.65 (0.85, 37.55)	0.07 ^#^	0.47 (0.19, 0.74)	0.001 *
**Model B ^b^**	4.33 (0.71, 26.53)	0.11	0.56 (0.19, 0.92)	0.003 *
**Model C ^c^**	4.47 (0.74, 26.89)	0.10	0.57 (0.28, 0.86)	<0.0001 *
**Model D ^d^**	4.34 (0.73, 25.67)	0.11	0.55 (0.24, 0.87)	0.001 *

Log-binomial model was used to assess the effectiveness of β-glucan ointment and remission. * Significant results (i.e., *p* ≤ 0.05). ^#^ Marginally significant result (i.e., 0.05 < *p* < 0.10). ^a^ No adjustment; ^b^ Adjusted for ointment usage; ^c^ Adjusted for ointment usage and wound duration; ^d^ Adjusted for ointment usage, wound duration, and burn degree. RR, risk ratio; RD, risk difference; 95% CI, 95% confidence interval.

**Table 3:** The effectiveness of β-glucan ointment on cytokines secretion.

Models	MD (95% CI)	SMD (95% CI)	*p*	Partial *η*^2^
IL-4				
**Model A ^a^**	75.48 (48.88, 102.08)	2.44 (1.34, 3.51)	<0.0001 *	0.624
**Model B ^b^**	75.04 (47.56, 102.53)	2.37 (1.28, 3.42)	<0.0001 *	0.618
**Model C ^c^**	79.56 (50.53, 108.58)	2.44 (1.34, 3.51)	<0.0001 *	0.648
**Model D ^d^**	77.27 (44.73, 109.82)	2.21 (1.16, 3.24)	0.0001 *	0.596
IL-17				
**Model A ^a^**	0.29 (−8.88, 9.46)	0.03 (−0.78, 0.84)	0.95	0.000
**Model B ^b^**	0.38 (−9.07, 9.83)	0.04 (−0.78, 0.85)	0.93	0.000
**Model C ^c^**	0.32 (−5.78, 6.42)	0.05 (−0.76, 0.86)	0.91	0.001
**Model D ^d^**	−1.56 (−8.09, 4.96)	−0.23 (−1.05, 0.58)	0.62	0.015
IFN-γ				
**Model A ^a^**	−0.13 (−6.15, 5.90)	−0.02 (−0.83, 0.79)	0.97	0.000
**Model B ^b^**	1.03 (−4.85, 6.92)	0.16 (−0.66, 0.97)	0.72	0.007
**Model C ^c^**	−1.16 (−11.53, 9.20)	−0.10 (−0.91, 0.71)	0.82	0.003
**Model D ^d^**	−1.93 (−13.34, 9.47)	−0.16 (−0.97, 0.66)	0.72	0.007

ANOVA/ANCOVA was used to assess the effectiveness of β-glucan ointment on cytokine secretion. * Significant results (i.e., *p* ≤ 0.05). ^a^ Adjusted for baseline cytokine; ^b^ Adjusted for ointment usage; ^c^ Adjusted for ointment usage and wound duration; ^d^ Adjusted for ointment usage, wound duration, and burn degree. MD, mean difference (i.e., mean_β-glucan—_mean Stratamed, SMD, standardized mean difference; 95% CI, 95% confidence interval).

## Data Availability

Not applicable.
